# A methodology for enhancing implementation science proposals: comparison of face-to-face versus virtual workshops

**DOI:** 10.1186/s13012-016-0429-z

**Published:** 2016-05-06

**Authors:** Brigid R. Marriott, Allison L. Rodriguez, Sara J. Landes, Cara C. Lewis, Katherine A. Comtois

**Affiliations:** 1 Department of Psychological and Brain Sciences, Indiana University, 1101 E. 10th St, Bloomington, IN 47405 USA; 2 Department of Psychological Sciences, University of Missouri, 320 S. 6th St, Columbia, MO 65211 USA; 3 National Center for PTSD, VA Palo Alto Health Care System, 795 Willow Rd., PTSD334, Menlo Park, CA 94025 USA; 4 Department of Psychiatry, Division of Health Services Research, University of Arkansas for Medical Sciences, 4301 W. Markham St., #755, Little Rock, AR 72205 USA; 5 Department of Psychiatry and Behavioral Sciences, University of Washington at Harborview Medical Center, Box 359911, 325 Ninth Avenue, Seattle, WA 98104 USA

**Keywords:** Implementation, Workshop, Virtual, Grant writing, Acceptability, Mixed methods

## Abstract

**Background:**

With the current funding climate and need for advancements in implementation science, there is a growing demand for grantsmanship workshops to increase the quality and rigor of proposals. A group-based implementation science-focused grantsmanship workshop, the Implementation Development Workshop (IDW), is one methodology to address this need. This manuscript provides an overview of the IDW structure, format, and findings regarding its utility.

**Results:**

The IDW methodology allows researchers to vet projects in the proposal stage in a structured format with a facilitator and two types of expert participants: presenters and attendees. The presenter uses a one-page handout and verbal presentation to present their proposal and questions. The facilitator elicits feedback from attendees using a format designed to maximize the number of unique points made. After each IDW, participants completed an anonymous survey assessing perceptions of the IDW. Presenters completed a funding survey measuring grant submission and funding success. Qualitative interviews were conducted with a subset of participants who participated in both delivery formats. Mixed method analyses were performed to evaluate the effectiveness and acceptability of the IDW and compare the delivery formats. Of those who participated in an IDW (*N* = 72), 40 participated in face-to-face only, 16 in virtual only, and 16 in both formats. Thirty-eight (face-to-face *n* = 12, 35 % response rate; virtual *n* = 26, 66.7 % response rate) responded to the surveys and seven (15.3 % response rate), who had attended both formats, completed an interview. Of 36 total presenters, 17 (face-to-face *n* = 12, 42.9 % response rate; virtual *n* = 5, 62.9 % response rate) responded to the funding survey. Mixed method analyses indicated that the IDW was effective for collaboration and growth, effective for enhancing success in obtaining grants, and acceptable. A third (35.3 %) of presenters ultimately received funding for their proposal, and more than 80 % of those who presented indicated they would present again in the future. The IDW structure and facilitation process were found to be acceptable, with both formats rated as equally strong.

**Conclusions:**

The IDW presents an acceptable and successful methodology for increasing competitiveness of implementation science grant proposals.

## Background

Implementation science is an emerging field, generated by the need to close the science-to-practice gap. That is, implementation science is the study of strategies designed to integrate evidence-based practices into real-world settings [1]. While the National Institutes of Health’s (NIH) funding of implementation science research projects appears to be increasing, the funding continues to be minimal compared to the billions of dollars (approximately US$30 billion) spent on basic and efficacy research annually [2]. For example, in 2010, only US$270 million (about 1 % of total NIH funding) was awarded to health quality, dissemination, and outcomes research at the Agency for Healthcare Research and Quality [2], of which implementation science represents a small portion. This disproportionate rate of funding is set within the context of an already very challenging funding climate, with funding success rates for new grants from the NIH dropping from 18.7 % in 2008 to 15.9 % in 2014 [3]. As implementation science has its own terminology [4], strategies [5], measures [6], models [7], and outcomes [8], there is much to learn and few grantsmanship resources or support structures available to enhance the rigor and relevance of implementation science proposals. Indeed, a recent editorial in *Implementation Science* expressed specific interest in capacity-building interventions to advance the field and increase interest in implementation science [9].

By our count, only four ongoing training programs in implementation science (that also include a focus on proposal development) exist in the USA. The Training Institute for Dissemination and Implementation Research in Health (TIDIRH) is a 5-day post-graduate training institute that provides its participants with a foundation of knowledge on conducting implementation science research with the hopes that these individuals will advance the field [10]. Similarly, the Implementation Research Institute (IRI) is a 2-year fellowship, in which fellows attend the institute for 1 week each summer and receive individualized mentoring from an implementation research expert [11]. IRI provides an opportunity for the fellows to receive personalized mentorship on developing an implementation science research agenda and preparing an implementation science-related research grant proposal. However, both of these opportunities prove to be competitive, with TIDIRH only accepting 13 % of applicants its first year [10] and IRI only accepting 36 % of applicants during its first 3 years [11]. Similar to IRI, the Mentored Training for Dissemination and Implementation Research in Cancer (MT-DIRC) involves a 2-year fellowship aimed at training cancer control researchers in implementation science research that includes a summer training program and ongoing, evidence-informed mentoring [12]. The Annual Academy Health Conference on the Science of Dissemination and Implementation in Health provides a brief, focused Technical Assistance workshop on grants, but it occurs only once a year and involves a one-time unstructured one-on-one meeting with a mentor following a didactic workshop [13]. Finally, the Colorado Research in Implementation Science Program (CRISP) hosted a one-time 2-day training workshop that included a half-day small-group consultation with implementation science experts on project proposals [14]. These are all excellent avenues for enhancing implementation science research funding success [10, 11, 14]; however, they require travel to the institute or conference, occur only once a year, and do not satisfy the demand (e.g., [10, 11]).

An alternative was developed by the Society for Implementation Research Collaboration (SIRC; formerly the Seattle Implementation Research Conference funded by a National Institute of Mental Health conference grant) to help fill this gap. The SIRC Implementation Development Workshop (IDW) is based on the Behavioral Research in Diabetes Group Exchange (BRIDGE) model [15] put forth by the Psycho-Social Aspects of Diabetes research group which facilitates expert feedback on “work in development.” The IDW is a research meeting open to members of the SIRC Network of Expertise—an invited contingent of established and new investigators, students, and evidence-based practice champions whose collective interest is in advancing the science and practice of implementation. These events are hosted either face-to-face at the SIRC biennial conference or virtually via web-based teleconferencing software (e.g., Adobe Connect, Zoom) with the ultimate goal of enhancing the likelihood that proposals are funded by external bodies, such as the NIH, Patient-Centered Outcomes Research Institute (PCORI), and the US Department of Veterans Affairs’ Quality Enhancement Research Institute (QUERI). The IDW has two further goals based on the facilitated discussion: share current and innovative ideas about implementation science and build collaboration between implementation scientists. Workshops that have utilized this type of model have not only gained important research insights but also led to funding, presentations, and publications [14].

Previous research examining the important features of group-based workshops revealed relational links between group members, such as group cohesiveness, trust, openness, participatory equality, and satisfaction with outcomes, as critical for effective information exchange [16]. With respect to group-based workshop platforms, in a comparison of face-to-face versus virtual teams of workers, Warkentin et al. [16] found that face-to-face teams demonstrated more satisfaction with the group interaction process, but levels of communication effectiveness were the same between face-to-face and virtual teams. Importantly, virtual communities of practice in business have displayed enhanced knowledge sharing and decreased social isolation by eliminating geographical barriers [17]. Thus, for group-based workshops, such as the IDW methodology, it is important to establish which format (face-to-face or virtual) is superior with respect to acceptability and effectiveness.

This manuscript presents the Implementation Development Workshops (IDWs) held by SIRC as a methodology for enhancing implementation science research grant funding success and evaluates the differential acceptability and effectiveness achieved by virtual versus face-to-face formats. This study is guided by two aims: (1) to explore the effectiveness and acceptability of the IDWs and delineate which features may enhance these desired outcomes and (2) to compare the effectiveness and acceptability of the face-to-face versus virtual IDWs and identify which components might make one format more effective or acceptable than the other.

## Methods

### Intervention methodology

#### Implementation Development Workshops (IDWs)

Each IDW is open to members of the SIRC Network of Expertise. Members can attend the IDW by registering in response to an emailed announcement about an upcoming IDW. During the registration process, a member can indicate if they would like to present or just attend and provide feedback. Those interested in presenting are asked to submit a short description of their project (~100 words). Presenters are selected based on several considerations including: their project’s implementation science relevance, timeline for submission, and stage of readiness to benefit from feedback. The facilitators are typically SIRC officers (or students of SIRC officers) who are trained in the IDW format and process and who have previously attended a mock IDW or actual IDW. Approximately a dozen attendees, multiple presenters (depending on the format), one facilitator, and two note takers attend each IDW. Presenters are typically researchers interested in receiving feedback on a proposal in development. The total number of presenters depended on the time allotted to the meeting (2–6 h), with 45 min devoted to each presentation regardless of meeting length. An attendee’s role consists of listening to the project presentations and providing verbal and written feedback to the presenter, and a presenter’s role involves providing a brief project overview and presenting the group with questions for which they would like solutions. The designated facilitator manages time, as well as coordinates and guides discussion and feedback. Finally, the note takers scribe all of the feedback attendees offer regarding the project proposal, so that presenters can stay engaged with the attendees without concern of missing or forgetting the feedback.

Each IDW begins with a brief orientation by the facilitator and introduction of everyone in attendance followed by an orientation to the workshop format. Then, each presenter is given 45 min. The presenter is asked to share a brief overview of their proposal or project in development for the first 10–20 min; leaving the remaining 25–35 min for feedback from attendees. Project presentations do not allow for technology, such as PowerPoint. Instead, based on the BRIDGE model [15], the verbal presentation is supplemented only by a one-page handout and a questions page. The questions page consists of three questions posited by the presenter on specific aspects of the proposal on which they want the attendees to focus their feedback, although discussion outside of these identified issues is permitted. These materials are sent to attendees at least 24 h prior to the virtual IDW or given to attendees at the beginning of the face-to-face IDW.

Also based on the BRIDGE model [15], following the project presentation, the facilitator coordinates the feedback from attendees in a particular manner. When attendees have feedback to provide, they raise their hand until acknowledged by the facilitator. The facilitator takes note of their names in the order that hands are raised and during the feedback section calls on the attendees in that order. Attendees are asked to only give one point of feedback per turn. If attendees have multiple points of feedback, they need to raise their hand again and wait to be called on to provide another point of feedback; attendees can thus raise their hand multiple times in succession. The project discussion continues as long as attendees have feedback to provide or until the 45-min mark. Because not everyone always has a chance to give verbal feedback on all of the presenter’s questions, the questions page provided with the handout has space for written responses that can be communicated during or at the end of the discussion. At the end of the project discussion, the facilitator directs the group to write any additional feedback they have, and then these question pages are collected and provided to the presenters as well as the notes from the session.

#### Face-to-face versus virtual format

The different formats for implementing the IDW resulted in some variations in procedure. The face-to-face IDW takes place once every 2 years at the biennial SIRC conference, while the virtual IDWs take place approximately three times a year, preceding the NIH tri-annual submission cycle. Given the format, the face-to-face IDW allows for all attendees to be in the same room and sit around a table. In the virtual IDW, only the individual presenting or the facilitator is viewable via a web camera. The individuals in the face-to-face IDW physically raise their hand, whereas the attendees of the virtual format click a “raise hand” button. The overall length of the IDW has differed by format as well. The face-to-face IDWs have lasted up to 6 h with six presenters, while the virtual IDWs last for only 2 h and have two presenters. Each presenter receives 45 min to provide an overview and receive feedback, regardless of the IDW format.

### Study design

The current study employed a simultaneous mixed methods program evaluation design. Data collection and analyses for the quantitative and qualitative data occurred concurrently to illuminate the effectiveness and acceptability of the IDW, any differences between the two formats, and features of the IDW and formats that made them more or less effective and acceptable. Acceptability was based on the definition put forward by Proctor et al. [8], which defines acceptability as “the perception among implementation stakeholders that a given treatment, service, practice, or innovation is agreeable, palatable, or satisfactory.” The quantitative data consisted of two surveys—an IDW program evaluation survey and a follow-up funding survey. The IDW program evaluation survey measuring attendees’ and presenters’ perceptions of their experience, including perceived acceptability and effectiveness of the IDW, was administered to all attendees and presenters and collected immediately following each IDW. A follow-up funding survey was later added to the evaluation to assess longer-term grant submission and funding outcomes, and this was sent to all presenters 6 months to 4 years after presenting a proposal at an IDW. This time gap allowed us to gather information about some grants that were submitted more than once and funded. In addition, qualitative interviews with a subset of IDW attendees and presenters were conducted. These semi-structured interviews focused on gaining more detail on the participants’ insight on group cohesiveness, the interaction process, and the facilitation process. All research procedures were approved by the Indiana University Institutional Review Board.

### Participants

Between October 2011 and January 2015, 72 network of expertise (NoE) members have participated in an IDW, with 40 members having participated in only a face-to-face IDW, 16 in only a virtual IDW, and 16 in both formats. The quantitative survey results consisted of 38 participants (face-to-face *n* = 12, 35 % response rate; virtual *n* = 26, 66.7 % response rate) for the program evaluation survey and 17 of the 36 presenters (face-to-face *n* = 12, 42.9 % response rate; virtual *n* = 5, 62.5 % response rate) for the follow-up funding surveys. All NoE members who participated in an IDW were invited to participate in an individual interview. Of the 11 interviews completed (response rate of 15.3 %), only seven were included in this study’s analyses to allow for comparison of participants’ experiences of both the face-to-face and virtual formats (as these participants had attended both).

### Quantitative data collection and measures

#### IDW program evaluation survey

After each IDW, participants were asked to fill out an anonymous 16-item IDW program evaluation survey, either via an in-person hardcopy survey or via a web-based survey for virtual IDWs. The evaluation survey inquired about both the attendees’ and presenters’ IDW experience. The items assessed whether the participant felt they learned, could apply what they learned, and had a firmer grasp of the principles and methods of implementation research. In addition, participants rated the effectiveness of various aspects of the format of the IDW, such as limiting the use of presentation technology, the role of the facilitator, the length of the workshop, and its acceptability. These items were rated on a 5-point Likert scale of 1 “Strongly Disagree” to 5 “Strongly Agree.” Table 1 lists all 16 items and provides descriptive statistics for each item.

**Table 1:** Evaluation survey means and standard deviations (*N =* 38)

Question	Face-to-face (*N* = 12) *M (SD)*	Virtual (*N* = 26) *M (SD)*	Total (*N =* 38) *M (SD)*
Effectiveness—Collaboration/Growth			
I learned things I did not know before.	4.58 (0.52)	4.50 (0.51)	4.53 (0.51)
I think I can apply a lot of what I learned in my own work.	4.50 (0.52)	4.42 (0.64)	4.45 (0.60)
I believe I have a firmer grasp of the principles and methods of implementation research.	4.00 (0.43)	3.77 (0.71)	3.84 (0.64)
Acceptability			
I was bored for a lot of the day.^a^	4.64 (0.67)	4.62 (0.57)	4.62 (0.59)
I found the discussions confusing.^a^	4.58 (0.90)	4.27 (0.67)	4.37 (0.75)
Facilitation/process			
*Structure*			
The day was well-organized.	4.75 (0.45)	4.85 (0.37)	4.82 (0.39)
Limiting the use of technology was helpful to get to the issues. *(Note: Limiting technology included no PowerPoint presentation in either format).*	4.50 (0.80)^**^	3.65 (0.89)^**^	3.92 (0.94)
The facilitator played a key role in maximizing the benefit for each presenter.	4.36 (0.67)	4.38 (0.80)	4.38 (0.76)
I thought a more free-flowing discussion with less facilitation would have been more effective.^a^	4.42 (0.67)	3.88 (0.95)	4.05 (0.90)
The written handout materials were helpful.	4.58 (0.67)	4.50 (0.65)	4.53 (0.65)
I appreciated the opportunity to respond to the questions in writing when I didn’t get a chance to verbally.	4.00 (0.74)	4.12 (0.73)	4.08 (0.72)
I would have preferred to have the presenter information at least 2 days in advance to prepare.	2.67 (1.37)	3.00 (1.30)	2.89 (1.31)
*Time*			
I would have preferred to hear more than 6 (2 for virtual) presentations/discussions.	2.08 (1.31)	2.08 (.94)	2.08 (1.05)
I would have preferred to hear fewer than 6 (2 for virtual) presentations/discussions.	1.73 (0.47)	1.62 (0.57)	1.65 (0.54)
The day was too long.	1.75 (0.62)^*^	1.31 (0.47)^*^	1.45 (0.56)
Having a break between each talk would have been better for me.	2.55 (0.69)^*^	2.04 (1.04)^*^	2.19 (0.97)

1 = Strongly disagree, 2 = Disagree, 3 = No opinion, 4 = Agree, 5 = Strongly Agree

^a^reverse scored

*Note.* ^*^ *p* < 0.05; ^**^ *p* < 0.01

#### Follow-up funding survey

A nine-item follow-up funding survey was sent to IDW presenters 6 months to 4 years after presenting to ask about whether their proposal was ultimately funded and how helpful the IDW was for enhancing their proposal’s competitiveness. Two items evaluated the utility of the feedback provided by the IDW attendees and the notes of feedback, using a 5-point Likert scale of 1 “Very Unhelpful” to 5 “Very Helpful”. Three items measured how much the presenter modified their grant proposal using the feedback provided by attendees at the IDW, believed the IDW feedback impacted the funding of his or her proposal (if funded), and believed the IDW feedback made their proposal more competitive (if unfunded) on a scale of 1 “None” to 5 “All of it/Completely.” Two additional items asked the presenter how many times they have submitted the proposal and how many of these submissions occurred after the IDW. Finally, two items inquired if the proposal presented was ultimately funded with response options of “Yes”, “No”, and “Not yet funded, plan to resubmit” and based on their experience, whether or not the presenter would present another grant proposal at a future IDW. Table 2 lists all nine items and descriptive statistics for each item.

**Table 2:** Follow-up funding survey means, standard deviations, and frequencies (*N =* 17)

Question	Face-to-face *(N* = 12)	Virtual (*N* = 5)	Total (*N* = 17)
Effectiveness—enhance success in obtaining grants	*M (SD)*	*M (SD)*	*M (SD)*
How helpful was the feedback given by the IDW attendees?	4.17 (.94)	4.40 (.55)	4.24 (.83)
Did you modify your grant proposal at all using the feedback given by attendees at the IDW?	2.25 (.75)	2.60 (.89)	2.35 (.79)
	*N (%)*	*N (%)*	*N (%)*
How many times have you submitted this proposal you presented?			
0	4 (33.3 %)	0 (0 %)	4 (23.5 %)
1	8 (66.7 %)	3 (60 %)	11 (64.7 %)
2	0 (0 %)	2 (40 %)	2 (11.8 %)
How many of these submissions occurred after the IDW?			
0	3 (25 %)	0 (0 %)	3 (17.6 %)
1	9 (75 %)	4 (80 %)	13 (76.5 %)
2	0 (0 %)	1 (20 %)	1 (5.9 %)
Was your proposal ultimately funded?			
Yes	4 (33.3 %)	2 (40 %)	6 (35.3 %)
No	5 (41.7 %)	2 (40 %)	7 (41.2 %)
Not yet funded, plan to resubmit	3 (25 %)	1 (20 %)	4 (23.5 %)
	*M (SD)*	*M (SD)*	*M (SD)*
If your proposal was funded, how much do you believe the IDW feedback impacted funding of your proposal?	2.50 (.58)	3.50 (.71)	2.83 (.75)
If your proposal was not funded, how much do you believe the IDW feedback made your proposal more competitive?	2.29 (.95)	2.50 (.71)	2.33 (.87)
Acceptability	*N (%)*	*N (%)*	*N (%)*
Based on your experience, would you present another grant proposal at a future IDW?			
Yes	10 (83.3 %)	5 (100 %)	15 (88.2 %)
No	2 (16.7 %)	0 (0.%)	2 (11.8 %)
Structure/facilitation process	*M (SD)*	*M (SD)*	*M (SD)*
How helpful was it having the notes of feedback sent to you?	4.33 (.49)^*^	3.20 (.84)^*^	4.00 (.79)

*Note.* ^*^ *p* < 0.05

### Qualitative data collection and measures

#### Semi-structured individual interviews

All attendees and presenters were recruited to participate in an individual interview via email. In order for an individual to agree to participate, they needed to provide informed consent according to Indiana University Institutional Review Board approved procedures. The participant then completed a 45-min phone-based individual interview. The interview included demographic questions about age, gender, degree level (e.g., Master’s degree, PhD), current position (e.g., student, researcher), and history of implementation funding as a principal investigator. A semi-structured interview guide was used to ask participants about their experience attending IDWs, their satisfaction with various aspects of the process and outcomes of the IDWs, and their perspectives on the relative benefits and disadvantages of the virtual and face-to-face IDW formats. A portion of the interview questions asked about the participant’s perspectives on group cohesiveness, trust, openness, and participatory equality based on Warkentin et al’s [16] study comparing face-to-face versus virtual teams collaborating on workplace tasks. The interviews were conducted by the first author (BRM) who was trained by the fourth author (CCL). All interviews were audio recorded and transcribed in order to be analyzed.

### Quantitative data analysis

Quantitative analyses were performed using SPSS 23 with two-tailed tests and alpha set at 0.05. Descriptive statistics were run to examine the overall effectiveness and acceptability of the IDW for both surveys. Exploratory analyses were conducted to assess the normality of the explanatory and response variables in the study. The Kolmogorov-Smirnov statistic showed a violation of the assumption of normality for these variables. Consequently, to compare the two IDW formats, a nonparametric test, the Mann–Whitney *U* Test, was utilized.

### Qualitative data analysis

Qualitative transcripts were entered into ATLAS.ti version 7.5.6. The research team reviewed transcripts to identify broadly emerging themes and developed a codebook based on these themes and relevant literature [16, 17]. The research team defined codes using a consensus process before coding began, and then revised the codebook throughout the coding process following an iterative coding design. The second and third authors (ALR, SJL) coded all interviews collaboratively, resolving any discrepancies as coding proceeded. All transcripts were then double-coded for accuracy. The research team met to discuss qualitative themes and proceed with qualitative analyses.

### Mixed methods analysis

The study results were analyzed using a QUAN + QUAL mixed methods approach, in which quantitative and qualitative results built upon one another (i.e., connect) so as to reach a more broadly informed understanding of the data (i.e., complementarity) [18]. Complementarity was utilized to evaluate the IDW and the two formats. In order to guide analyses, the aims of the study were subdivided into four sets of mixed methods research questions. (1) Is the IDW effective for collaboration and growth? What makes it effective or what impacts effectiveness? (2) Does the IDW enhance success in obtaining grants? How does it enhance success in obtaining grants? (3) Is the IDW acceptable and/or satisfying? What about the IDW makes it acceptable/satisfying? and (4) Is the IDW structure and facilitation process acceptable and/or satisfying? What about the structure and facilitation process makes it acceptable and/or satisfying?

## Results and discussion

### Participants

Due to the anonymous nature of both quantitative surveys and their primary purpose being that of program evaluation, no demographic data was collected. For the qualitative interviews, participants were predominantly female (*N* = 6, 85.7 %) with an average age of 37 (*SD* = 5.74). Participants ranged from trainee (*N* = 1, 14.3 %) to mid/senior career level (*N* = 1, 14.3 %), but were primarily junior level faculty (e.g., assistant professor) (*N* = 5, 71.4 %). Nearly three-quarters of the participants had a history of implementation science-focused funding as a principal investigator (*N* = 5, 71.4 %).

### Mixed methods results and discussion

Quantitative results for the post-workshop evaluation survey are presented in Table 1, and quantitative results for the funding survey are presented in Table 2. After performing quantitative and qualitative analyses as described above, mixed methods results were considered in the context of four sets of research questions, presented and answered in Table 3.

**Table 3:** Mixed methods results displaying complementarity (*N =*7)

Quantitative	Qualitative
Q1: Is the IDW effective for collaboration and growth? Yes, overall, both face-to-face and virtual IDW participants indicated positive agreement that they learned something they did not know, thought they could apply a lot of what they learned in their own work, and believed they had a firmer grasp of the principles and methods of implementation research.	Q1: What makes it effective or what impacts effectiveness? Qualitative participants indicated that in both face-to-face and virtual formats, they received thoughtful and helpful feedback from experts in the field, and interactions were positive and professional. While participants indicated that both formats facilitated making connections with colleagues, they also indicated that in-person IDWs offered more options for networking and socializing than virtual IDWs.
Q2: Does the IDW enhance success in obtaining grants (effective)? Yes, the IDWs were effective with many presenters both submitting the grant proposals they presented and successfully receiving funding for those proposals.	Q2: How does it enhance success in obtaining grants? Participants reported that the IDW offered concrete ideas for revising their proposals and that hearing how others viewed the project helps them to reframe their own thoughts. Many responded that they incorporated feedback received at the IDW and that subsequent proposals were funded. A few participants also noted that the objective of the IDW (i.e., to provide feedback on a proposal rather than a completed project) was an effective tool for the purpose of obtaining better funding outcomes.
Q3: Is the IDW acceptable/satisfying? Yes, participants indicated that they were not bored and did not find discussions confusing. They also agreed that they would be interested in presenting proposals at future IDWs.	Q3: What about the IDW makes it acceptable/satisfying? Participants indicated that the online platform was easy to use, and that the structured process of both the in-person and virtual IDW made it easy to participate. Most participants expressed a preference for the in-person IDW, however, satisfaction with outcomes did not seem to differ in substantial ways between formats. The benefit of senior colleague/expert involvement was mentioned as an element that made the experience particularly satisfying.
Q4: Is the IDW structure and facilitation process acceptable and/or satisfying? Yes, participants agreed in both face-to-face and virtual IDWs that the structure of the IDW and facilitation process was effective and/or acceptable. See Table 1 for detailed items. Only four differences were found between formats. Face-to-face IDW participants rated the idea that limiting the use of technology was helpful to get to the issues, that the day was too long, and having a break between talks would be better higher than virtual participants. Face-to-face IDW presenters also found the notes of feedback more helpful than the virtual presenters did.	Q4: What about the structure and facilitation process makes it acceptable and/or satisfying? Participants cited many aspects of the IDW as very helpful, including note-taking, three questions, limited technology, and the role of the facilitator. The single comment rule was also seen as helpful for managing discussion, though a few participants mentioned concerns that this rule may have limited depth of the discussion. Participants also indicated that although doing more presentations during the in-person IDWs was tiring, it makes sense to do this given the costs associated with in-person meetings.

### Question Set 1: Is the IDW effective for collaboration and growth? What makes it effective or what impacts effectiveness?

Growth was defined as an increase or development in the areas of knowledge (e.g., new learning) or professional related outcomes (e.g., new research skills). Overall, participants in both formats indicated positive endorsement that they learned something they did not know, thought they could apply a lot of what they learned in their own work, and believed they had a firmer grasp of the principles and methods of implementation research (see Table 1). During qualitative interviews, participants confirmed that the IDW was effective in terms of collaboration and growth, regardless of format. Some participants mentioned the networking opportunity the IDWs provided.

“…The new people that I didn’t know…I was excited to get to have known them. … Their comments were so thoughtful and insightful, [and I thought], ‘I’m going to look up their work’. It was actually kind of stimulating that way for me, professionally. I just branched out a little bit and got to know some other people who are doing really fantastic work in the field.”

“…Since I had met those people, I was actually able to go back and get other feedback from them later. So it kind of gave me a way to access folks that maybe I hadn’t had before.”

Although virtual IDWs were still viewed as effective resources for collaboration and growth, a few participants mentioned that it was easier to make these connections and network during face-to-face meetings.

“It’s just sort of a little increased networking opportunity there with the in-person…That’s probably the piece you don’t get online.”

In both formats, participants appreciated the presence of senior colleagues during discussions.

“When you have a nice mix of people and then you have these really fabulous experts who provide insightful, and constructive, and well-articulated feedback, it makes for a super interesting IDW.”

### Question Set 2: Does the IDW enhance success in obtaining grants? How does it enhance success in obtaining grants?

Follow-up funding survey results revealed that the IDWs were effective with regard to both submitting grant proposals and funding success. Of the participants who replied to the survey, 82.4 % submitted the proposal presented at the IDW for grant funding. Of those who submitted, 35.3 % ultimately received funding and 26.7 % plan to resubmit the proposal (indicative of receiving a strong score from the review committee). On average, presenters believed the feedback given in the IDWs impacted the funding of their proposal “Some” to “Quite a bit” (*M =* 2.83, *SD =* 0.75). Although 41.2 % of presenters did not ultimately receive funding for the proposal presented, the presenters still believed the feedback provided at the IDW made their proposal, on average, “Some” to “Quite a bit” more competitive (*M* = 2.33, *SD =* 0.87).

Qualitative results echoed the sentiments of the quantitative surveys with regards to enhancing the funding success, and participants indicated that they made revisions to their proposals after receiving expert feedback at the IDW. One participant said,

“For me, it was really helpful, so I actually ended up changing my project, based on the feedback, and that was the grant that I submitted that was funded.”

Participants also praised the effectiveness of the IDW objective to provide feedback for projects before they were submitted.

“I really liked it. …normally at conferences… you present completed work. Even when people have helpful, critical feedback, it’s too late. You’re like, ‘That’s a great idea, and I don’t have a time machine.’ I think what’s really cool is to have a way, especially in a field that’s a new field, for people actually to get that critical feedback at a point in time when they can actually do something about it. … this is having a structured, recurring way to get that kind of feedback.”

### Question Set 3: Is the IDW acceptable and/or satisfying? what about the IDW makes it acceptable/satisfying?

According to survey results, participants were satisfied with the IDW and found it acceptable. Participants indicated that they were not bored or confused throughout the day. In addition, results from the funding survey also indicated the IDW to be acceptable to the presenters, with 88.2 % of presenters agreeing that they would present another grant proposal at a future IDW. Qualitative results revealed that although participants had a preference for face-to-face meetings, both formats were deemed acceptable and satisfying, especially considering the relative costs and benefits of each format.

“Overall I would say I think that the value of doing it online outweighs any drawbacks relative to face-to-face.”

“I don’t think it would have changed how I felt, I just personally prefer being face-to-face.”

Despite these findings, a few participants indicated that they were more easily distracted in the virtual IDWs than they were during face-to-face meetings, due in part to the differences in anonymity. One participant mentioned:

“I noticed that some… names dropped off the list of participants during the call… I don’t think that would happen that people would just stand up and walk out in the middle of in an in-person thing.”

A few participants also indicated that active participation was greater for some attendees at face-to-face meetings than during virtual meetings; however, this did not seem to have a negative effect on learning.

“I learn things just listening, even if I don’t participate much. I learn about, ‘Oh, that’s a good way to think about that.’”

### Question Set 4: Is the IDW structure and facilitation process acceptable and/or satisfying? What about the structure and facilitation process makes it acceptable and/or satisfying?

Participants in both formats agreed that the day was well-organized and not too long (see Table 1). Participants from both groups also agreed that the written materials provided (i.e., the one-page overview of each project/proposal) were helpful and that responding to issues in writing (i.e., providing feedback on the presenter’s questions) was helpful, and the facilitator was also seen as crucial to the success of the IDW. A statistically significant difference was found between the formats for the item: “Limiting the use of technology was helpful to get to the issues” (*U* = 75, *z* = 2.67, *p* = .01, *r* = 0.43). Participants in the face-to-face format (*M* = 4.50, *SD* = 0.80) indicated a higher level of agreement with this statement than virtual format participants (*M* = 3.65, *SD* = 0.89), though this may be confounded because the virtual format is inherently based in technology. Presenters at the face-to-face IDW also found the notes of feedback sent to them after the IDW more helpful than the virtual presenters did, *U* = 8, *z* = 2.62, *p* = .01, *r* = 0.64, which may be because attendees in the virtual format were less likely to take notes and send them on to the organizers. In addition, statistically significant differences were observed for the item “The day was too long” (*U* = 96, *z* = 2.18, *p* = 0.03, *r* = 0.35) and the item “Having a break between each talk would have been better for me” (*U* = 87, *z* = 2.02, *p* = .04, *r* = 0.33), with face-to-face format participants demonstrating more agreement for both items. Important to note is that the face-to-face IDWs were approximately three times longer than the virtual format (6 versus 2 h, respectively).

During the qualitative interviews, participants repeatedly expressed positive views regarding the effectiveness and acceptability of the IDW structure, and most of these positive views were consistent between formats. One participant expressed that the IDW structure served as an equalizer in this way, stating:

“The way that the interactions are facilitated… It was a pretty similar experience to be honest with you from the online versus face-to-face.”

Participants noted the helpfulness of various components of the structure:

“The note-taking was really helpful… Knowing that somebody was taking notes let me actually just listen and talk to people.”

“I liked that we didn’t have to create a Power Point, and we had the one-page thing. I feel like that just made it a lot easier just to have conversations.”

“[Generating three questions ahead of time] was useful… so that people could think about those three questions, but… by the time I got to the workshop, my questions had evolved, so it was a good thing that we were able to veer away from the questions if necessary during the feedback and workshop.”

“I thought it was very effective… [the facilitator was] very good about yielding the conversation or turning it to the person who had raised their hand … it kept things moving, so that it wasn’t one person talking continuously.”

A few participants saw the IDW structure served to overcome certain barriers, particularly barriers to the virtual setting:

“The nice thing is, with the facilitation process, you don’t have to look to see when someone might be finished to start talking. … Having a facilitation process makes the non-verbal cues not as important, or maybe not important at all.”

“I think the online atmosphere worked well with this type of structure, with the special way that it’s facilitated. Because everything is moderated and modulated and that works well on the internet where things can be or when you're on the phone where it can be tricky when people start to talk over each other.”

“As for the facilitation, I really liked it, and I think that’s part of what helped everything feel equal… Like you just raised your hand and your name was put down, and it was in that order. So it wasn’t like you had to interrupt someone who was a senior person to get a word in edgewise.”

However, there were a few drawbacks to the structure of the IDW. One participant said:

“Because of the way the facilitation works, that there’s one comment and then we move directly into a different comment that is likely to be entirely different in content, the interaction between attendees is minimal within the process itself. Then on top of that, we didn’t do video introductions of the attendees at all [in the virtual format], and so there was not really much of a networking component to this activity.”

### Limitations

The results from this preliminary investigation of the IDW methodology are promising, though the results should be considered within the context of limitations due to the quantitative data being obtained from program evaluation surveys. For instance, the quantitative data for the face-to-face IDW only included the evaluation forms for one of two face-to-face IDWs that had taken place at the time the data analyses were performed. Moreover, due to the evaluation’s anonymous nature, we were not able to look at differences in those who participated in both formats versus only one format or differences between presenters versus attendees. Also, response rates for the individual interviews (15.3 %), the evaluation survey (35 % for face-to-face and 66.7 % for virtual format), and follow-up funding survey (42.9 % for face-to-face and 62.5 % for virtual format) were low, although they reflect similar response rates to evaluations of other trainings [14]. We strategically designed the IDW procedures to target members of the SIRC network of expertise to achieve the aim of enhancing grant funding competitiveness through quality feedback; however, because of this, the generalizability of the findings beyond an expert group warrants exploration. Finally, the funding surveys were not administered in a consistent time frame for presenters and this may have had an impact on whether or not they had submitted, received notification regarding funding, and/or resubmitted.

### The future of implementation development workshops

Feedback from the quantitative surveys and qualitative interviews have been and will continue to be incorporated into the redesign of subsequent IDWs. For example, the length of the most recent face-to-face IDW was reduced to three and half hours from 6 hours, based on feedback. Although both delivery formats were found to be equally effective and acceptable, two caveats of the virtual format revealed by qualitative analyses were that networking was more difficult and attendees were less accountable (e.g., would leave the call). Future IDWs should incorporate methods for increasing networking opportunities within the virtual format (e.g., use a program that allows for all attendees to share their videos simultaneously) and emphasizing the importance of not leaving the IDW until it has completed. In addition, the IDWs had previously been focused solely on research proposals, but the mission of the IDW has recently been modified and expanded to encompass evidence-based practice champions and intermediaries as well as researchers and to provide feedback for both research grant and/or practical implementation project proposals.

SIRC is committed to supporting IDWs for the foreseeable future. SIRC recently became a society requiring paid membership to support its capacity to sustain initiatives such as the IDW that aim to advance implementation science through rigorous methodology and evaluation. SIRC Officers retain responsibility for hosting virtual IDWs that precede the NIH submission cycle and solicit volunteer attendance from established investigators on the SIRC advisory board to ensure a quorum for the event. SIRC has recently partnered with the Australasian Implementation Conference organizers to host an IDW at their third biennial event October 2016. Given the demand for the IDW coupled with the rather routinized and efficient procedure established by the SIRC Officers, the IDW remains a priority and a feasible initiative to sustain.

## Conclusions

This study revealed that the IDWs held by SIRC represent a strong methodology for enhancing implementation science grant proposals. The IDW format was effective in terms of both increasing collaboration and growth among its participants and grant funding success and acceptable to both attendees and presenters. Similar to Warkentin et al. [16], the virtual format was found to be equally as effective and acceptable as the face-to-face format. This is promising as the use of virtual formats can greatly reduce the geographic barriers that often emerge for and are associated with face-to-face formats [17]. With the current funding climate and an increasing demand for frequently offered and accessible grantsmanship resources and support structures, the IDW presents a unique avenue for increasing the quality and rigor of implementation science proposals and advancing the field.

## Abbreviations

BRIDGE: Behavioral Research in Diabetes Group Exchange
CRISP: Colorado Research in Implementation Science Program
IDW: Implementation Development Workshop
IRI: Implementation Research Institute
NIH: National Institutes of Health
NoE: Network of Expertise
PCORI: Patient-Centered Outcomes Research Institute
QUERI: Quality Enhancement Research Institute
SIRC: Society for Implementation Research Collaboration
TIDIRH: Training Institute for Dissemination and Implementation Research in Health
